# A Community Based Study on the Mode of Transmission, Prevention and Treatment of Buruli Ulcers in Southwest Cameroon: Knowledge, Attitude and Practices

**DOI:** 10.1371/journal.pone.0156463

**Published:** 2016-05-26

**Authors:** Jane-Francis K. T. Akoachere, Frankline S. Nsai, Roland N. Ndip

**Affiliations:** Department of Microbiology and Parasitology, Faculty of Science, University of Buea, Buea, Cameroon; IPBS, FRANCE

## Abstract

**Background:**

Buruli ulcer (BU) is a neglected tropical disease affecting the skin, tissues and in some cases the bones, caused by the environmental pathogen *Mycobacterium ulcerans* (*M*. *ulcerans*). Its mode of transmission is still elusive. Delayed treatment may cause irreversible disabilities with consequent social and economic impacts on the victim. Socio-cultural beliefs, practices and attitudes in endemic communities have been shown to influence timely treatment causing disease management, prevention and control a great challenge. An assessment of these factors in endemic localities is important in designing successful intervention strategies. Considering this, we assessed the knowledge, attitude and practices regarding BU in three endemic localities in the South West region, Cameroon to highlight existing misconceptions that need to be addressed to enhance prompt treatment and facilitate effective prevention and control.

**Methods and Findings:**

A cross-sectional study was executed in three BU endemic health districts. Using qualitative and quantitative approaches we surveyed 320 randomly selected household heads, interviewed BU patients and conducted three focus group discussions (FGDs) to obtain information on awareness, beliefs, treatment, and attitudes towards victims. The influence of socio-demographic factors on these variables was investigated.

**Results:**

Respondents (84.4%) had a good knowledge of BU though only 65% considered it a health problem while 49.4% believed it is contagious. Socio-demographic factors significantly (P<0.05) influenced awareness of BU, knowledge and practice on treatment and attitudes towards victims. Although the majority of respondents stated the hospital as the place for appropriate treatment, FGDs and some BU victims preferred witchdoctors/herbalists and prayers, and considered the hospital as the last option. We documented beliefs about the disease which could delay treatment.

**Conclusion:**

Though we are reporting a high level of knowledge of BU, there exist fallacies about BU and negative attitudes towards victims in communities studied. Efforts towards disease eradication must first of all target these misconceptions.

## Introduction

Buruli ulcer (BU) is a disease of the skin, underlying tissues and in some cases the bones, caused by *Mycobacterium ulcerans*, an environmental pathogen belonging to the family of bacteria that cause tuberculosis and leprosy.BU is the third most common *Mycobacterium* infection of immune-competent hosts after tuberculosis and leprosy, and it is the most poorly understood of the three diseases [1]. BU evolves in three stages. It begins with the pre ulcerative phase which is characterized by a firm, non-tender nodule and sometimes plaques or oedema. The second phase involves ulceration of the skin, causing osteomyelitis as a possible complication. In the third phase, a granulomatous healing response takes place followed by fibrosis, scarring, calcification and contractures, with the possibility of permanent disabilities [2].

Virulence of *M*. *ulcerans* is due to the production of a toxin, mycolactone, which destroys the subcutaneous adipose tissue resulting in the development of large ulcers [3]. Although the disease can affect any part of the body, it has been found to affect mostly the extremities (particularly the limbs) [4].

Prompt diagnosis and appropriate treatment is necessary to prevent progression of the disease to the severe form which causes irreversible physical disabilities [5] that have social and economic impacts on the victim. Although rational use of antibiotic is important, surgery is the standard treatment, which at early stages of the disease, involves only an excision. For larger lesions with extensive necrosis of the skin, surgery removes necrotic tissue to stop the spread of the infection. Grafting may be done and in certain cases, amputation carried out.

WHO [2] recommends combination therapy for treatment with daily intramuscular streptomycin and oral rifampicin for 8 weeks for all stages of the disease. BCG is the only vaccine available for prevention of BU though there has been conflicting reports on its effectiveness. Portaels [6] reported the protective effect of neonatal BCG vaccination against severe forms of BU disease. However, a recent study [7] has reported no significant evidence of protection of routine BCG vaccination on the risk of developing either BU or severe forms of the disease and suggests comprehensive studies using different existing strains of BCG.

Although *M*. *ulcerans* may be acquired from the natural environment, its environmental reservoirs and exact mode of transmission remain elusive. Aquatic bugs have been suggested as reservoirs of the pathogen and are believed to be involved in transmission [8]. A recent study [9] reported the persistence of *M*. *ulcerans* in underwater decaying organic matter in a water source for BU patients suggesting underwater decaying organic matter may serve as a reservoir. Transmission is believed to occur by direct infection of skin, lesions or vector associated following a skin prick or bite. However, human-human transmission has not been established. Risk factors associated with infection include poor wound care and failure to wear protective clothing or shoes during farming, swimming or wading in contaminated water and a low level of education [10–13]. Outbreaks have been associated with ecological changes such as damming or flooding [14].

BU disease has been identified by the WHO as an emerging Neglected Tropical Disease (NTD) of the poor. It has been reported as a cause of morbidity in 33 countries in Africa, Asia, the Americas and Western Pacific with Africa being the worst affected region [2]. The disease has a high prevalence in poor and rural areas where healthcare services are limited. In Africa, countries in West and Central Africa including Benin, Ghana, Ivory Coast, Cameroon and the Democratic Republic of Congo have been reported to have a high burden [2].

The socioeconomic burden of the disease resulting from delay in treatment has been well described [15–18]. Treatment of severe cases requires prolonged hospitalization with a concomitant increase in cost and impoverishment contributing to social isolation of patients [16]. Individuals with permanent disabilities have suffered from reduced marriage prospects or faced divorce, stigmatization and participation restrictions which may affect school attendance for children [19].

An understanding of the socio-cultural factors in endemic localities is very important in the success of public health interventions to improve treatment outcomes, disease prevention and control. In endemic parts of Africa, there is a wide range of cultural beliefs, attitudes, behaviors and practices regarding BU that have influenced the health seeking behavior of patients hence delaying treatment, contributing to the progression of the disease to severity [20–22].

BU was first described in Cameroon in 1969 in individuals from the Nyong River valley in the Center Region, between the villages of Ayos and Akonolinga, which has predominantly the equatorial forest [23]. The disease has since then remained endemic in this area and has also been reported in other geographic regions of the country including the Adamawa, East, Far North, and the South West Regions [24, 25] during national BU surveys. Of the BU foci in Cameroon, extensive research has generated clinical, ecological, entomological, epidemiological, anthropological and socio-economic data on BU in Ayos and Akonolinga (where several NGOs have partnered with the Ministry of Health to carry out interventions and conduct research), and Bankim in the Adamaoua region [4, 9, 11,16, 25–27]. In the Nyong River basin, the local name for BU, “Atom” [26] has a very negative connotation (victim of an evil eye). Perception of the disease as having a mystical aetiology in this locality has contributed to delayed treatment of some victims [22].

Although Cameroon is one of the most endemic countries in Central Africa, there is little or no information on the disease in the South West. Unlike Akonolinga and Ayos, no NGO has focused on BU disease control in any of the endemic localities in the South West region. Therefore, information on the knowledge, attitude and practices (KAPs) related to the disease in this locality is very important as this will inform policy that could enhance early treatment and minimize the physical, economic and social impacts of the disease. This study therefore assessed the KAPs of inhabitants in some BU endemic localities in the South West Region of Cameroon.

## Materials and Methods

### Study setting and design

The study was carried out in Mbonge, Ekondo-Titi and Muyuka, which are the health districts (HDs) reporting the majority of BU cases in the South west region of Cameroon.

**Fig 1:**
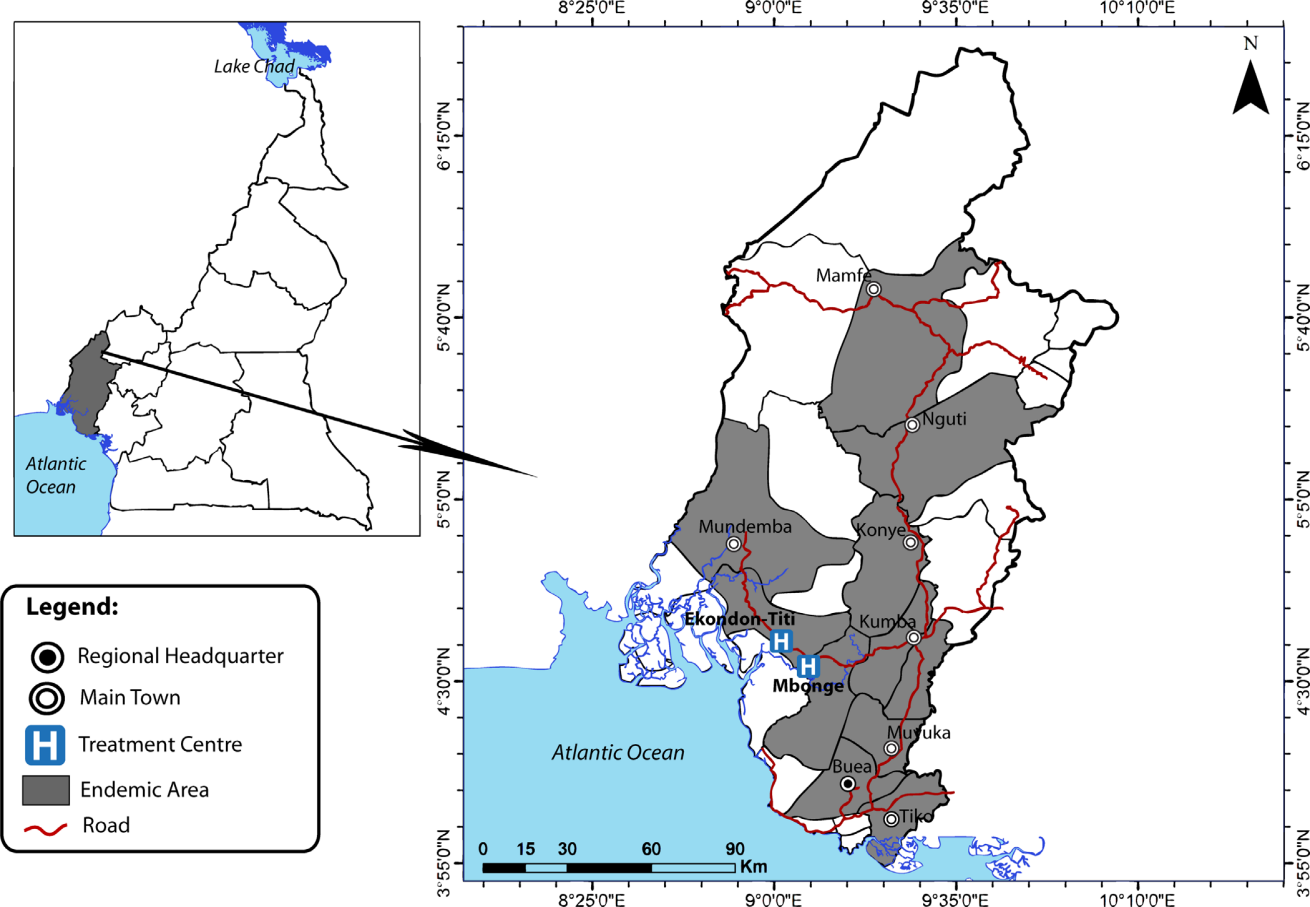
Map of Study Area.

Mbonge and Ekondo Titi with an approximate population of 73,500 and 78,700 inhabitants respectively are bordered in the North West by Mundemba and in the East by Kumba (Fig 1). Characterized by lowlands and slow moving water bodies, these two HDs usually record high temperatures (approximately 28°C) during the rainy season and even higher (about 31°C) in the dry season. The main occupation of inhabitants of these localities is farming and most of them are plantation farmers. The only two treatment centers for BU in the South West region are found in Mbonge and Ekondo-Titi. The Mbonge treatment center was set up in 2006. In 2012, the Ekondo-Titi treatment center became operational.

Muyuka on the other hand is found towards the southern part of the region and is bordered in the far south by Buea and Tiko (Fig 1). Muyuka has an approximate population of 95,850 inhabitants. This area has alternating high and low lands, and is also characterized by relatively high temperatures both in the rainy and the dry season. The main occupation of inhabitants is farming. Their farmlands and environments are characterized by marshy lands and slow moving water bodies. The environmental and occupational characteristics of the inhabitants expose them to BU.

This study was a cross sectional survey involving 320 participants, in which their perceptions, attitudes and practices on BU were investigated. Data was obtained quantitatively and qualitatively through structured questionnaires, interviews and focus group discussions.

### Questionnaires

Questionnaires that had been piloted and validated were administered to household heads of affected communities. This questionnaire had three sections: demographics, understanding of causes of the disease and treatment seeking behavior of patients, and community practice and attitude towards the BU disease sufferers (See S1 Text). To facilitate data collection, two community health workers per health district were trained on how to administer the questionnaires and they assisted in this study.

### Focus group discussions

A total of 3 FGDs were carried out, one in each HD. To this effect, 6–10 community members (See S1 Table) were put together, depending on how available participants were and discussions were initiated by the investigator. This was done to obtain in-depth information about their understanding of the cause and treatment seeking behavior of patients as well as their attitudes and practices on BU. A tape recorder was used to record discussions.

### Interviews

BU patients were identified and interviewed verbally in order to know how they managed their condition and how they were treated by community members.

#### Sampling technique

Various households were selected using a simple random sampling technique. Advantage was taken of household numbering for the distribution of Long Lasting Insecticide Nets (LLINs) in 2011, and using a table of random numbers, houses were picked at random. Sampling was done without replacement in order to eliminate the chances of a given household being chosen more than once. Numbers were picked till the required sample size was attained.

### Ethical Considerations

Ethical clearance for this study was obtained from the Institutional Review Board of the Faculty of Health Sciences, University of Buea. Administrative clearance was obtained from the South West Regional Delegation of Public Health. At the study sites, permission was sought from the district medical officers. All participants were informed about the objectives of the study and their enrollment in the study was voluntary. When required, questions were asked in ‘Pidgin English’ (or local language) and completion of questionnaire done by research assistant. Study participants signed an informed consent form to indicate their willingness to participate in the study.

### Data analysis

The statistical package EPI Info 7 was used to design the questionnaire template and to enter data in this study. SPSS version 20.0 statistical software was used in data cleaning, management and analysis. A descriptive analysis on the cases was done. The relationship between study outcome (the dependent variables) and the independent variables (demographic factors) was analyzed using the chi-square test. Unadjusted logistic regression analyses were carried out to determine the best prediction of the dependent variable from several demographic variables.

## Results

### Demographic characteristics of respondents

The communities surveyed had more male (64.7%) than female (35.3%) headed households (Table 1). The highest number of respondents was in the age group 21–40 (49.1%). Most of them were Christians (94.7%), married (76.6%), farmers (51.9%) and had resided in their respective communities for more than 5 years (61.6%). Individuals with secondary education comprised 44.1% of study population (Table 1).

**Table 1:** Demographic characteristics of community members and their understanding of BU.

Demographic Characteristics	N (%)	Knows what BU is (%)		Have a local name they call BU (%)		Is BU a Health Problem (%)		Can BU be transmitted from one person to another (%)	
Total	320(100)	No	Yes	No	Yes	No	Yes	No	Yes
		15.6	84.4	80.9	19.1	35	65	50.6	49.4
**Sex**		***X***^**2**^ ***= 1*.*958*,**	***P = 0*.*162***	**X**^**2**^ ***= 1*.*828*,**	***P = 0*.*176***	**X**^**2**^ ***= 2*.*158*,**	***P = 0*.*142***	***X***^**2**^ ***= 0*.*176*,**	***P = 0*.*675***
Female	113(35.3)	19.5	80.5	80.9	19.1	41.6	58.4	52.2	47.8
Male	207(64.7)	13.5	86.5	78.7	21.3	31.9	68.1	49.8	50.2
**Age**		**X**^**2**^ ***= 5*.*564*,**	***P = 0*.*135***	**X**^**2**^ ***= 1*.*572*,**	***P = 0*.*666***	**X**^**2**^ ***= 9*.*567*,**	***P = 0*.*023***	**X**^**2**^ **= 2.651,**	**P = 0.449**
<20	1(0.3)	100	0	100	0	100	0	100	0
21–40	157(49.1)	15.3	84.7	83.4	16.6	34.4	65.6	47.1	52.9
41–60	140(43.8)	15.0	85.0	78.6	21.4	33.6	66.4	52.9	47.1
61^+^	22(6.9)	18.2	81.8	77.3	22.7	63.6	36.4	59.1	40.9
**Education**		**X**^**2**^ ***= 0*.*759*,**	***P = 0*.*859***	**X**^**2**^ **= 6.878,**	**P = 0.076**	**X**^**2**^ **= *21*.*324*,**	***P = 0*.*000***	**X**^**2**^ ***= 4*.*726*,**	***P = 0*.*193***
No Education	38(11.9)	13.2	86.8	65.8	34.2	44.7	55.3	63.2	34.2
Primary	122(38.1)	15.6	84.4	82.0	18.0	48.4	51.6	55.7	44.3
Secondary/High School	141(44.1)	17.0	83.0	84.4	51.6	55.7	44.3	82.0	18.0
University	19(5.9)	10.5	89.5	78.9	21.1	5.3	94.7	57.9	42.1
**Religion**		**X**^**2**^ ***= 0*.*270*,**	***P = 0*.*874***	**X**^**2**^ ***= 9*.*214*,**	***P = 0*.*010***	**X**^**2**^ ***= 7*.*797*,**	***P = 0*.*020***	**X**^**2**^ ***= 1*.*030*,**	***P = 0*.*597***
Christian	303(94.7)	15.5	84.5	81.2	18.8	35.0	65.0	50.2	49.8
Muslim	13(4.1)	15.4	84.6	92.3	7.7	46.2	53.8	53.8	46.2
Traditional	4(1.3)	25.0	75.0	25	75	100	0	75	27
**Occupation**		**X**^**2**^ ***= 6*.*347*,**	***P = 0*.*096***	**X**^**2**^ ***= 6*.*698*,**	***P = 0*.*090***	**X**^**2**^ ***= 10*.*757*,**	***P = 0*.*013***	**X**^**2**^ ***= 11*.*256*,**	***P = 0*.*010***
Farming	166(51.9)	13.9	86.1	75.9	24.1	35.5	64.5	47.0	53.0
Trading	51(15.9)	19.6	80.4	88.2	11.8	47.1	52.9	54.9	45.1
Professional/Admin	72(22.5)	11.4	88.6	87.5	12.5	23.6	76.4	44.4	55.6
Other	31(9.7)	29.0	71.0	80.6	19.4	51.6	48.4	77.4	22.6
Marital Status		**X**^**2**^ ***= 2*.*654***	***P = 0*.*448***	**X**^**2**^ ***= 8*.*236*,**	***P = 0*.*04***	**X**^**2**^ **= *0*.*452*,**	***P = 0*.*929***	**X**^**2**^ ***= 1*.*682*,**	***P = 0*.*641***
Divorced	9(2.8)	33.3	66.7	88.9	11.1	44.4	55.6	33.3	66.7
Married	245(76.6)	15.5	84.5	82.4	17.6	35.9	64.1	51.0	49.0
Single	54(16.9)	14.8	85.2	79.6	20.4	35.2	64.8	53.7	46.3
Widowed	12(3.8)	8.3	91.7	50.0	50.0	41.7	58.3	41.7	58.3
**Time Spent in the community**		***X***^**2**^ ***= 2*.*958*,**	***P = 0*.*228***	***X***^**2**^ ***= 9*.*222*,**	***P = 0*.*010***	***X***^**2**^ ***= 5*.*598*,**	***P = 0*.*061***	***X***^**2**^ ***= 0*.*347*,**	***P = 0*.*841***
Less than 1yr	24(7.5)	8.3	91.7	87.5	12.5	33.3	66.7	54.2	45.8
Between 1yr and 5yrs	98(30.6)	20.4	79.6	89.8	10.2	45.9	54.1	52.0	48.0
More than 5yrs	197(61.6)	14.2	85.8	75.6	24.4	32.0	68.0	49.2	50.8

### Awareness of Buruli ulcer and its causes

Of the surveyed population, 84.4% knew BU, with 19.1% of them having a local name for the disease. In Mbonge and Ekondo Titi, the disease is called ‘Oroto’ whereas in Muyuka the local name is ‘Bolingo’, both names meaning ‘unhealing wound’. Sixty-five percent (65%) regarded BU as a health problem, and 49.4% believed BU could be transmitted from person to person (Table 1). Analysis with respect to demographic characteristics gave more insights about awareness on BU, and its causes. Respondents of age 61years and above were more likely to think that BU is not a health problem (χ^2^ = 9.567, P = 0.023) (Table 1). With respect to sex there was no significant difference between males and females in their level of awareness of BU. Participants with tertiary education were more likely to regard BU as a health problem (χ^2^ = 21.324, P = 0.000). Participants with traditional religion were less likely to see BU as a health problem (χ^2^ = 7.797, P = 0.020), and more likely to have a local name for BU (χ^2^ = 9.214, P = 0.010). Professionals/administrators were more likely to think that BU is a health problem (χ^2^ = 10.757, P = 0.013) as expected but surprisingly, believed that it could be transmitted from one person to another (χ^2^ = 11.256, P = 0.010) (Table 1). Widows (χ^2^ = 8.236, P = 0.04) and participants that have spent more than 5 years in the community (χ^2^ = 9.222, P = 0.010) were more likely to have local names for BU.

Respondents expressed diverse opinions about the aetiology of BU (Table 2). Approximately 59% did not know the cause of BU. Among those that thought they knew, 14.1% thought it was due to bites by worms in water ponds and swampy areas, 6.6% attributed the disease to witchcraft or insect bite, 5.3% to poor hygiene while only 4% attributed BU to bacterial infection. Among the participants who stated the cause of BU, stratification with respect to level of education revealed a significant difference (χ^2^ = 57.855, P = 0.001), in their understanding of the causes of BU (Table 2).

**Table 2:** Understanding of the causes of BU by community members with respect to level of education.

Education	What causes BU (X^2^ = 57.855, P = 0.001)							
	Don't know (%)	Worms (%)	Poor hygiene (%)	Witchcraft (%)	Mut mut fly (%)	Insects (%)	Snake bite (%)	Bacterial infection (%)
No Education	52.6	18.4	0.0	10.5	2.6	5.3	7.9	2.6
Primary	69.7	11.5	4.9	8.2	2.5	3.3	0.0	0.0
Secondary/High school	51.4	16.4	7.9	5.0	3.6	10.0	0.0	5.0
University	57.9	5.3	0.0	0.0	5.3	5.3	0.0	26.3
Total	58.9	14.1	5.3	6.6	3.1	6.6	1.3	4.1

Information obtained from FGDs complemented the data obtained through the structured questionnaires. FGD participants attributed it to water or marshy areas, or witchcraft while others believed BU was of mystical origin. FGD also had mixed opinions about the infectiousness of the disease as some people thought it could be transmitted while others believed it was not contagious. However the majority of the FGD participants saw BU as a health problem. Some of their opinions on the causes of BU included:

✓ *One can get* BU *when he/she gets exposed to ‘lamba’ (marshy areas) or riverine areas (Female*, *Ekondo-Titi);*
✓ *My uncle went for a death ceremony and there he was bewitched*. *Since then he has been suffering with this illness for 8 years now (Female*, *Muyuka);*
✓ *This kind of illness is not of a natural cause (Male*, *Muyuka);*
✓ *My cousin’s wife has been suffering for two years now with BU*. *He thinks his wife has been bewitched by her parents (Male*, *Mbonge);*

Among the BU patients interviewed, some did not know the cause of the disease but recognized it as a health problem, while some thought it was due to witchcraft as seen from this response:

*When I was sick with* BU, *my aunt told me that I have been bewitched through the bite of a snake and that its fangs are inside my wound (Male*, *Muyuka)*.

### Knowledge and practice of study partcipants on treatment of Buruli ulcer

**Fig 2:**
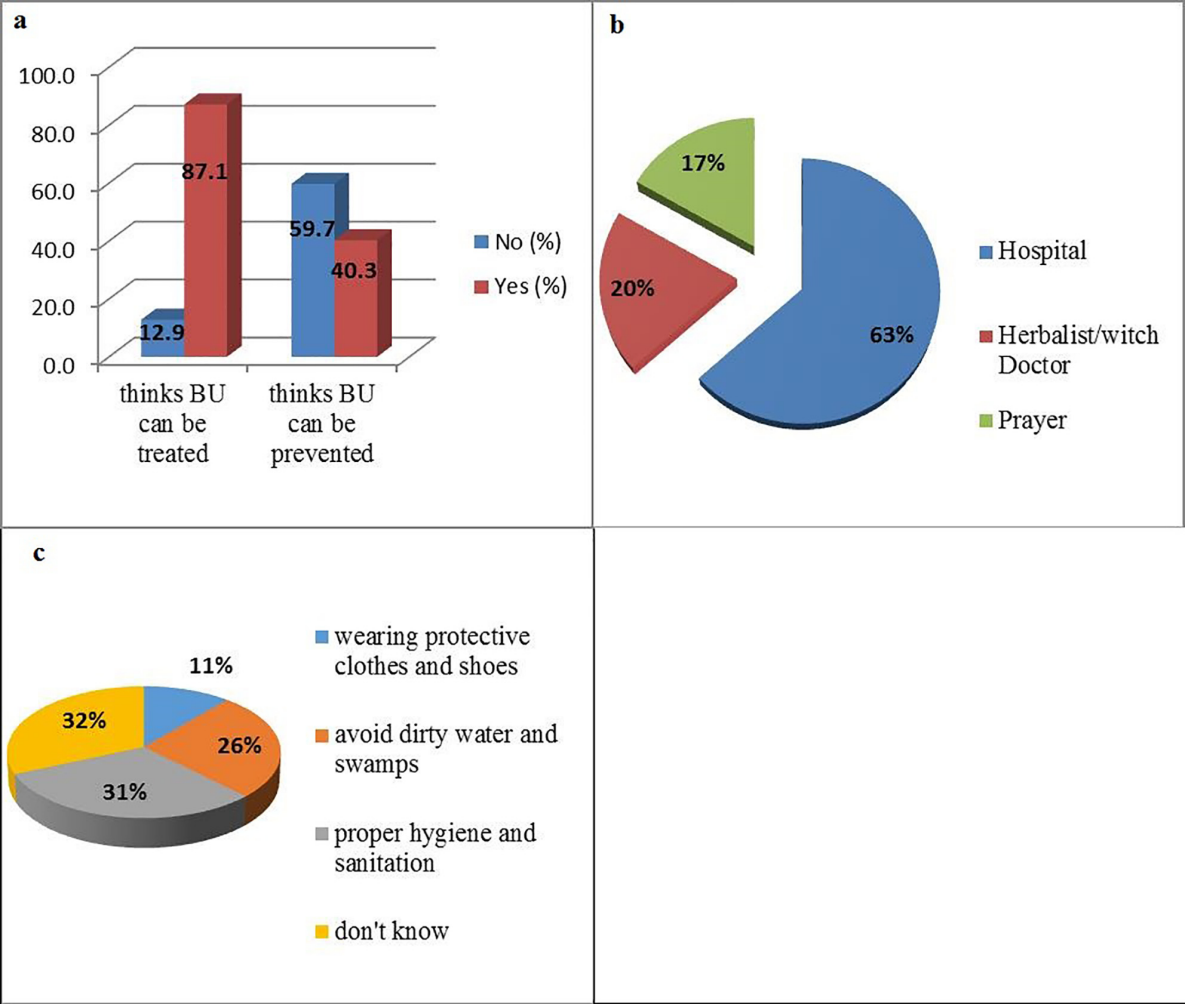
**Knowledge and practice of study partcipants on treatment of BU** (a) Graph showing proportion of respondents and their opinion on treatment and prevention of BU (b) Opinion on best means of treating BU (c) Preventive measures listed by participants

About 87% of the household heads admitted that BU can be treated while 59.7% thought it could be prevented (Fig 2A). However, respondents had varied opinions on where treatment could be obtained. While 63% of them indicated that BU can be treated at the hospital, 17% and 20% respectively reported prayers (miracles) and from Herbalists/witch doctors (Fig 2B).

FGD participants mentioned herbalists/witchdoctor as places consulted by BU patients for treatment since some strongly believed witchcraft was the cause of the disease, and that some patients considered medical treatment only when they realized they could not be treated by traditional healers. Below are some of the statements from FGD participants on treatment seeking behavior:

✓ *My uncle was initially convinced that his condition (BU) could be handled by traditional healers*, *until they failed him*. *That is when he went to the hospital for treatment (Female*, *Muyuka)*.
✓ *I remember a foreigner that lived with BU for some time and was convinced that only traditional healers could help him until he finally died (Male*, *Mbonge)*.

Reasons given by FGD participants for patients seeking treatment from traditional healers included inferiority complex, the high cost of medical treatment and purported inability of the hospital to manage the condition due to prolong hospitalization, high cost of transportation and other expenses, as seen by these statements:

✓ *In my community*, *some members are infected but don’t want to go to the hospital for treatment because they are ashamed of their condition*. *They think it is witchcraft and also believe treatment is expensive (Male*, *Ekondo-Titi)*.
✓ *I think consulting a witchdoctor is cheaper when compared with treatment at the hospital*. *Some of these families cannot afford transport and other expenses to get their patients treated (Female*, *Muyuka)*.
✓ *Consulting the witchdoctor is always the last resort as usually*, *the hospital is unable to treat the patient (Female*, *Mbonge)*

Opinions of patients were similar to those of FGD participants. While some patients said they could be treated only by a witchdoctor/herbalist, others strongly believed in biomedicine:

✓ *We have tried to seek treatment from the hospital to no avail; we have also consulted herbalists who are not capable of treating me*. *We have thus resorted to consulting a witchdoctor to enquire if I have been cursed by somebody or if it is due to witchcraft*. *So we have tried all possible options just to make sure I am ok*, *but still we found no solution (Female*, *Mbonge)*.
✓ *Since they say a spiritual snake (totem) is the cause of my wound*, *I believe a special witchdoctor can help remove the fangs of the snake that has been planted in my leg before the wound will start getting healed (Male*, *Ekondo-Titi)*
✓ *Despite the fact that I have been grafted three times within the past three years of my stay in this hospital without any success*, *I still believe that I am going to be treated here (Male*, *Mbonge)*.

Among the reasons given by patients for seeking help from traditional healers were the long duration of treatment in the hospital which made them to think that treatment was ineffective and knowledge of someone successfully treated by a traditional healer as shown below:

✓ *I have been told of a patient who had this illness and was treated by a witchdoctor so I think he is going to treat me as well because I have been directed to him*. *In addition*, *I have been in this hospital for over 5 months now but my wound keeps increasing so I think the witchdoctor is going to help me (Female*, *Mbonge)*

Preventive measures of the disease reported by respondents included proper hygiene and sanitation (31%), avoidance of dirty water and swamps (26%), and wearing of protective clothes and shoes (11%); however up to 32% had no idea of any preventive measure (Fig 2C).

### Attitude and perceptions of community members towards BU patients

About 67% of the respondents had seen someone with BU. While 70.3% of them thought that BU patients could be regarded as normal people in the society, only 41% would allow their children or family members to interact freely with BU patients. Over half of the respondents (57.8%) stated that there are traditional beliefs attributed to BU while almost the same proportion (57.5%) accepted that patients should be allowed to go to school or public places.

It was observed that community’s attitudes and practices towards patients were influenced by socio-demographic factors. Male participants would less likely allow their relatives to interact freely with BU patients (χ^2^ = 8.157, P = 0.004) (Table 3). Participants with no education would less likely regard BU patients as normal people in the society (χ^2^ = 22.657, P = 0.000), less likely allow their relatives to interact with BU patients (χ^2^ = 10.215, P = 0.017) and also less likely accept that they should be allowed to go to school or public places (χ^2^ = 19.264, P = 0.000). Farmers were more likely to have seen someone with BU (χ^2^ = 9.844, P = 0.020) but less likely to regard them as normal people in the society (χ^2^ = 15.846, P0.001), less likely to allow their relatives to interact freely with them (χ^2^ = 11.868, P = 0.008) or to accept that they should be allowed to go to public places (χ^2^ = 8.005, 0.046) (Table 3). Individuals with traditional religion were more likely to have seen someone with BU (χ^2^ = 6.803, P = 0.033), less likely regard BU patients as normal people in the society (χ^2^ = 7.957, P = 0.019), less likely accept that they should be allowed to go to public places (χ^2^ = 7.648, P = 0.022) but were more likely to accept that there are traditional beliefs attributed to BU (χ^2^ = 5.101, P = 0.078) (Table 3).

**Table 3:** Attitude and perception of community members towards BU patients.

Demographic Characteristics	N (%)	Have seen someone with BU before (%)		Thinks BU Patients are regarded as normal people in the society (%)		Will allow children or family members to interact freely with BU Patients (%)		Thinks there are traditional beliefs attributed to BU (%)		Thinks BU patients should be allowed to go to school or public places (%)	
Total	320(100)	No	Yes	No	Yes	No	Yes	No	Yes	No	Yes
		33.1	66.9	29.7	70.3	58.4	41.6	42.2	57.8	42.5	57.5
**Sex**		***X***^**2**^ ***= 0*.*020*,**	***P = 0*.*888***	**X**^**2**^ ***= 1*.*355*,**	***P = 0*.*244***	**X**^**2**^ ***= 8*.*157*,**	***P = 0*.*004***	***X***^**2**^ ***= 0*.*304*,**	***P = 0*.*581***	***X***^***2***^ ***= 1*.*414*,**	***P = 0*.*234***
Female	113(35.3)	33.6	66.4	25.7	74.3	47.8	52.2	44.2	55.8	38.1	61.9
Male	207(64.7)	32.9	67.1	31.9	68.1	64.3	35.7	41.1	58.9	44.9	55.1
**Age**		**X**^**2**^ ***= 2*.*534*,**	***P = 0*.*469***	**X**^**2**^ ***= 5*.*426*,**	***P = 0*.*143***	**X**^**2**^ ***= 1*.*989*,**	***P = 0*.*575***	**X**^**2**^ **= 2.381,**	**P = 0.497**	**X**^**2**^ **= 1.505,**	**P = 0.681**
<20	1(0.3)	100	0	0	100	100	0	100	0	0	100
21–40	157(49.1)	34.4	65.6	26.8	73.2	56.7	43.3	42.7	57.3	40.8	59.2
41–60	140(43.8)	32.1	67.9	30.0	70.0	60.0	40.0	42.9	57.1	43.6	56.4
61^+^	22(6.9)	27.3	72.7	50.0	50.0	63.6	36.4	31.8	68.2	50.0	50.0
**Education**		**X**^**2**^ ***= 5*.*253*,**	***P = 0*.*154***	**X**^**2**^ **= 22.657,**	**P = 0.000**	**X**^**2**^ **= *10*.*215*,**	***P = 0*.*017***	**X**^**2**^ ***= 2*.*108*,**	***P = 0*.*550***	**X**^**2**^ **= 19.264,**	**P = 0.000**
No Education	38(11.9)	21.1	78.9	55.3	44.7	76.3	23.7	31.6	68.4	65.8	34.2
Primary	122(38.1)	36.9	63.1	34.4	65.6	63.1	36.9	43.4	56.6	41.8	58.2
Secondary/High School	141(44.1)	31.2	68.8	22.0	78.0	51.1	48.9	43.3	56.7	41.8	58.2
University	19(5.9)	47.4	52.6	05.3	94.7	47.4	52.6	47.4	52.6	05.3	94.1
**Religion**		**X**^**2**^ ***= 6*.*803*,**	***P = 0*.*033***	**X**^**2**^ ***= 7*.*957*,**	***P = 0*.*019***	**X**^**2**^ ***= 2*.*954*,**	***P = 0*.*228***	**X**^**2**^ ***= 5*.*101*,**	***P = 0*.*078***	**X**^**2**^ **= 7.648,**	**P = 0.022**
Christian	303(94.7)	32.3	67.7	28.1	71.9	57.8	42.2	43.6	56.4	40.9	59.1
Muslim	13(4.1)	61.5	38.5	53.8	46.2	61.5	38.5	23.1	76.9	61.5	38.5
Traditional	4(1.3)	0	100	75.0	25.0	100	0	0	100	100	0
**Occupation**		***X***^***2***^ ***= 9*.*844*,**	***P = 0*.*020***	***X***^***2***^ ***= 15*.*846*,**	***P = 0*.*001***	***X***^***2***^ ***= 11*.*868*,**	***P = 0*.*008***	***X***^***2***^ ***= 1*.*324*,**	**P = 0.723**	***X***^***2***^ ***= 8*.*005*,**	***P = 0*.*046***
Farming	166(51.9)	26.5	73.5	38.0	62.0	66.9	33.1	41.6	58.4	50.0	50.0
Trading	51(15.9)	45.1	54.9	29.4	70.6	56.9	43.1	49.0	51.0	35.3	64.7
Professional/Admin	72(22.5)	33.3	66.7	12.5	87.5	45.8	54.2	38.9	61.1	33.3	66.7
Other	31(9.7)	48.4	51.6	25.8	74.2	45.2	54.8	41.9	58.1	35.5	64.5
**Marital Status**		***X***^***2***^ ***= 2*.*967***	***P = 0*.*397***	***X***^***2***^ ***= 5*.*015*,**	***P = 0*.*171***	***X***^***2***^ ***= 0*.*389*,**	***P = 0*.*942***	***X***^***2***^ ***= 2*.*420*,**	***P = 0*.*490***	***X***^***2***^ ***= 4*.*063*,**	***P = 0*.*255***
Divorced	9(2.8)	33.3	66.7	33.3	67.7	55.6	44.4	44.4	55.6	66.7	33.3
Married	245(76.6)	32.2	67.8	28.6	71.4	58.4	41.6	44.1	55.9	42.0	58.0
Single	54(16.9)	40.7	59.3	27.8	72.2	57.4	42.6	37.0	63.0	37.0	63.0
Widowed	12(3.8)	16.7	83.3	58.3	41.7	66.7	33.3	25.0	75.0	58.3	41.7
**Time Spent in the community**		***X***^**2**^ ***= 2*.*674*,**	***P = 0*.*263***	***X***^**2**^ ***= 0*.*307*,**	***P = 0*.*858***	***X***^**2**^ ***= 1*.*883*,**	***P = 0*.*390***	***X***^**2**^ ***= 3*.*913*,**	***P = 0*.*141***	***X***^***2***^ ***= 1*.*159*,**	***P = 0*.*560***
Less than 1yr	24(7.5)	45.8	54.2	25.0	75.0	62.5	37.5	58.3	41.7	33.3	66.7
Between 1yr and 5yrs	98(30.6)	35.7	64.3	29.6	70.4	63.3	36.7	44.9	55.1	40.8	59.2
More than 5yrs	197(61.6)	30.5	69.5	30.5	69.5	55.3	44.7	38.6	61.4	44.2	55.8

Information obtained from the non-parametric tests on attitude and perception of participants mentioned above (Table 3) was in concordance with multiple logistic regression analysis without adjusting for other variables as shown on Table 4. For participants with the opinion that BU patients should be regarded as normal people in the society, education [1.86 (1.27, 2.27), P<0.05] and religion [0.36 (0.15, 0.86), P<0.05] contributed significantly. Gender [0.49 (0.29, 0.83), P<0.05], occupation [1.30 (1.01, 1.69), P<0.05] and time spent in the community [1.38(1.05, 1.82), P<0.05] also contributed to the participants’ attitude of allowing their children and family members to interact with patients. For participants that agreed that there were traditional beliefs attributed to BU, religion contributed significantly [1.61 (1.14, 2.29), P<0.05] (Table 4). Level of education [1.61(1.14, 2.29), P<0.05] contributed significantly among those who agreed that BU patients should be allowed to go to public places.

**Table 4:** Unadjusted Odds Ratio (OR) (at 95% Confidence Interval) for acceptance of (BU) patients by community members against their socio-demographic variables.

Socio-demographic Variable	Have seen someone with BU before	Thinks BU patients should be regarded as normal people in the society	Will allow children or family members to interact freely with BU patients	Thinks there are traditional beliefs attributed to BU	Thinks BU patients should be allowed to go to school or public places
**Gender**	0.83 (0.49, 1.41)	0.74 (0.41, 1.32)	**0.49 (0.29, 0.83)**	1.24 (0.75, 2.06)	0.79 (0.47, 1.34)
**Age**	1.06 (0.70, 1.60)	0.99 (0.64, 1.53)	1.11 (0.73, 1.67)	1.08 (0.72, 1.60)	1.10 (0.74, 1.64)
**Education**	0.97 (0.68, 1.39)	**1.86 (1.27, 2.27)**	1.44 (1.01, 2.04)	0.87 (0.62, 1.23)	**1.61 (1.14, 2.29)**
**Marital Status**	0.97 (0.62, 1.51)	0.85 (0.53, 1.34)	0.93 (0.59, 1.47)	1.38 (0.88, 2.16)	1.21 (0.78, 1.88)
**Occupation**	0.78 (0.60, 1.00)	1.23 (0.92, 1.65)	**1.30 (1.01, 1.69)**	1.14 (0.88, 1.46)	1.13 (0.87, 1.46)
**Religion**	0.83 (0.27, 1.80)	**0.36 (0.15, 0.86)**	0.54 (0.20, 1.43)	**3.36 (1.07, 10.52)**	0.30 (0.11, 0.82)
**Time spent in the community**	1.07 (0.82, 1.40)	1.06 (0.79, 1.43)	**1.38(1.05, 1.82)**	1.18 (0.91, 1.52)	0.96 (0.74, 1.25)

Values in bold indicate significant difference at P<0.05 OR (lower value, upper value)

**Fig 3:**
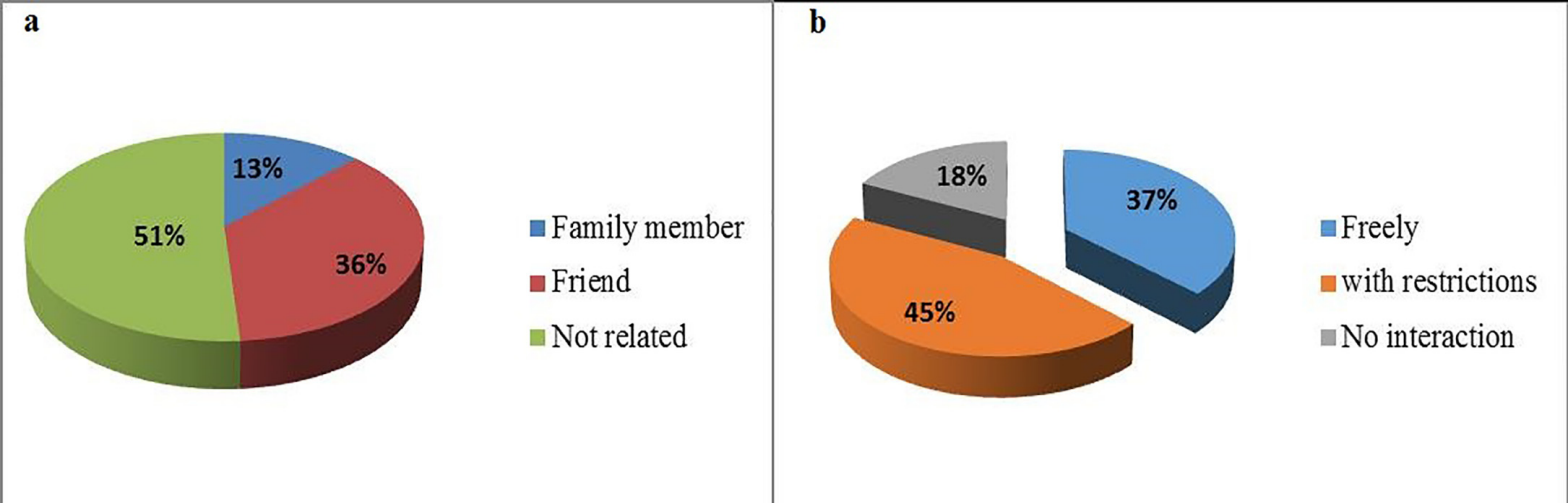
**Relationship with, and attitude towards BU patients** (a) Relationship with patient (b) Level of interaction with patients.

Fifty-one percent (51%) of participants that had seen BU patients were not related to these BU patients, 36% of them were friends to the patients and 13% were family members of these patients (Fig 3A). Among those that had seen BU patients, 45% would interact with them with restrictions, while 37% would interact with them freely. However, 18% never interacted with a BU patient (Fig 3B).

Participants of the FGDs equally demonstrated reservation and discrimination towards BU patients with the following comments:

✓ *I was not comfortable at all when I had to sit in the same vehicle with a BU patient*. *The situation got worst when the car had to stop for a while at a police check point*. *The wound had a terrible smell that made me and the other passengers feel so uncomfortable (Male*, *Mbonge)*.
✓ *It is practically impossible for us to give a post of responsibility to a BU patient*. *First because of his/her health condition and second because it is BU*. *Any person that sees the ulcer gets frightened instantly or disgusted by it and so people besides the patient will always feel uneasy (Male*, *Ekondo-Titi)*.
✓ *Someone with BU is always looked upon like an outcast*. *I can’t be in the same place with somebody like that for a while because of the horrible odour from the patient’s wound (Male*, *Mbonge)*.

Even though BU patients were rejected by some FGD participants, others had positive attitudes and perceptions towards sufferers as seen below:

✓ *Since I already know about this bad illness*, *I will welcome the patient and try to encourage him/her to seek treatment at the hospital*, *rather than thinking that it is due to witchcraft (Male*, *Ekondo-Titi)*.
✓ *Although other people felt terrible when they came to visit my father*, *I did not run away from him just because he had BU*. *He is still my father after all and he is completely healed now (Female*, *Muyuka)*.

BU patients in the study area have suffered isolation as prolonged hospitalization became a burden to the family. Others faced marital problems as the disease was attributed to witchcraft as seen below:

✓ *My marriage is at the verge of breaking down due to my condition*. *This is because my mother- and father- in-law think that I was bewitched by my own parents and have to go back to my village as I can only be treated with the help of my parents (Female*, *Mbonge)*
✓ *I have been here for over three years now and my family members have neglected me here*. *They think my condition is a burden to them as I have been unable to be treated for this while (Male*, *Mbonge)*.

**Fig 4:**
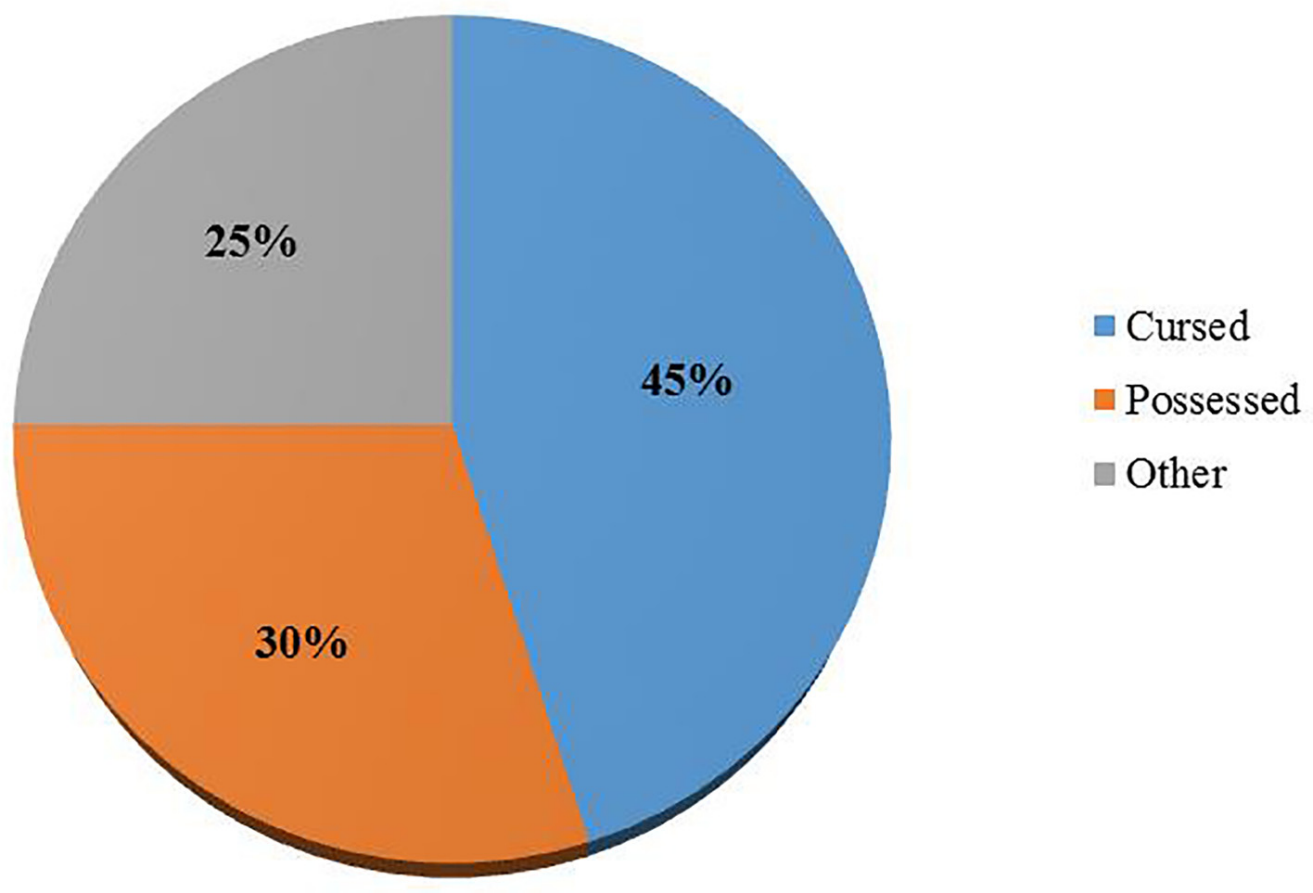
Traditional beliefs attributed to BU.

Among the respondents that admitted the existence of traditional beliefs attributed to BU, 45% of them were convinced it was due to a curse, 30% said the patients were possessed and about 25% attributed it to other beliefs like witchcraft and totems (snakes) (Fig 4).

Some participants of FGD attributed BU to witchcraft and other traditional beliefs. They stated:

✓ *My cousin was so convinced that his wife’s condition (BU) was due to witchcraft from her family*. *There is almost a break-up in their marriage due to his wife’s condition that has been attributed to witchcraft (Female*, *Ekondo-Titi)*.
✓ *I believe that if the patient is responsible for his/her condition*, *the wound will never get healed unless he/she confesses (Male*, *Mbonge)*.
✓ *There are some patients that when their wound is almost getting dry (healed)*, *it is refreshed by totems (snakes) at night through witchcraft*, *so it can never get healed (Male*, *Muyuka)*.

## Discussion

The management of BU has been a challenge in endemic areas because of poor knowledge and beliefs attributed to the disease which have greatly influenced disease treatment, prevention and control. Proper sensitization of these communities about the disease is very important but for this to be achieved, there is a need to understand the existing knowledge and perceptions of the disease, as well as attitude towards victims of the disease to be able to design intervention strategies that would give desired results. Our study, being the first in the South West region of Cameroon, assessed the knowledge, attitude and practices on the mode of transmission, prevention and treatment of BU in endemic localities.

### Awareness of BU

Our study revealed an overall high level of knowledge (84.4%) of BU in study area with local names like ‘Orotor’ and ‘Bolingo’ meaning ‘unhealing wound’ given to the disease describing its chronicity. Our findings contradict the results of Kamga *et al*. [28] who reported a low level of knowledge (42.1%) of BU in the South West region. In their study, health districts were randomly selected and could have included those not reporting the disease, hence the lower level of knowledge obtained. Our study focused on BU foci in the South West region. Our study indicates that the disease is well known in areas where it occurs, supporting the findings of Renzaho *et al*. [29], who reported a high level of awareness of the disease in an endemic area. The high level of awareness observed in the present study could be due to the rigorous nationwide population campaigns during the national BU survey in 2004 by the National BU Control Programme (NBUCP). Since then, community health workers continued with the sensitization. However, socio-demographic factors such as age, level of education, religion, and occupation significantly influenced the level of understanding of the disease by community members (Table 1). Over half (59%) of respondents did not know the exact cause of BU whereas 6.5% and 4% attributed it to witchcraft and bacterial infection, respectively. The rest of the participants attributed the cause of the disease to human and environmental factors such as poor hygiene, bite from worms in marshy areas, insect or snake bite. Some of these factors have been reported in literature as risk factors for BU [11, 13, 30]. An understanding of the causes of BU was significantly influenced by level of education of respondents (χ^2^ = 57.855, P = 0.001) (Table 2), with the majority of those linking the disease to a bacterial infection being respondents with tertiary level of education. Some FGD participants and BU victims believed the disease was of mystical origin or caused by witchcraft while some did not know the cause. Contrary to our findings, studies in other African countries with BU [20] have reported witchcraft as a major cause of the disease perceived by study participants. However, the percentage of our respondents (6.5%) that stated witchcraft as the cause of the disease ties with that (5.2%) reported by Renzaho *et al*. [29] in a BU endemic locality in Ghana. Notwithstanding, our findings show that witchcraft is still believed to be the cause of the disease in our study area.

Despite the fact that BU is well known, participants generally demonstrated a poor understanding of its aetiology and mode of transmission. Almost half (49.4%) of the participants thought that the disease could be transmitted from one person to another. This was significantly influenced by occupation (χ^2^ = 11.256, *P* = 0.010) and surprisingly, with professionals/administrators more likely to think that the disease is communicable. This is a misconception because human to human transmission of the disease has never been documented. Considering the disease as contagious could contribute to negative attitudes towards the patients. Again, more than half of respondents believed BU cannot be prevented (Fig 3A) showing that community sensitization on BU in study areas is insufficient and needs to be reinforced. However, preventive measures listed by those who knew it is preventable (Fig 3C) have been reported in the literature [10, 11, 13]. The NBUCP since its creation in 2002 depended on external partners for funding. From 2010 funding became a great challenge as external funding drastically reduced and the programme since then has been run with limited funds from the Cameroonian Government [31]. Because of this, control activities have greatly reduced and sensitization is now conducted as a component of campaigns against Neglected Tropical Diseases which is not as detailed as efforts focusing only on BU. This could explain the poor knowledge on aetiology and mode of transmission observed in our study.

### Treatment seeking behavior

In this survey, the majority of the respondents (87%) thought BU could be treated (Fig 2A). Among these, about 2/3 (63%) believed the hospital was the ideal place for seeking treatment (Fig 2B). This is consistent with the response of the survey in which very few (6.5%) believed that the disease was caused by witchcraft. Consulting the hospital to seek treatment is associated with illnesses that are perceived to be caused by natural factors while illnesses that are perceived to have been induced by sorcery are addressed by a traditional healer to counteract the sorcery [29]. Our findings show that treatment seeking behavior could be related to the perception of the cause of the disease. However, information from some FGDs and BU victims revealed that hospital treatment was their last option as most initially sought treatment either from herbalist or witchdoctors. The long duration of treatment of severe cases could have made some FGD participants and BU patients to regard hospital treatment as being ineffective.

The notion that the disease may be of mystical origin could have caused some respondents to consider prayers as the best option for treatment. Dependence on faith healers by BU patients has also been reported by Owusu-Sekyere *et al*. [32]. Our study shows that fallacies are some of the causes of delay in medical treatment which contribute to progression of the disease to severity further underscoring the need for intensive health education. In West Africa, where belief in sorcery is known to affect perceptions of illness and health care seeking behavior, an inverse correlation appears to exist between the prevalence of these beliefs and the extent of health education disseminated in the area [33].

Treatment of severe cases is not only long but involves increase in cost [17, 18] and this has led to isolation of patients in the hospital by their families [20]. This was confirmed by one of the BU victims who reported that he was abandoned by his family because he became a burden as he had spent three years in the hospital. Other reasons advanced by FGD participants that prevented patients from seeking treatment included amongst others being ashamed of the condition and high cost compared to treatment by a traditional healer.

In Cameroon, medical costs for BU treatment are covered by international humanitarian aid non-governmental organizations. However, direct medical costs are incurred by patients during periods of stock outs [16] as treatment centers are located in remote areas. The recent reduction in funding of the NBUCP has further increased direct medical costs. Non-medical direct costs such as transportation and miscellaneous costs or indirect costs associated with treatment [16] could have also contributed to high cost. The South West Region has just two treatment centers for BU, which are located in Ekondo Titi Health District and Mbonge Health District respectively. These centers are located far away from other health districts, such as Muyuka which usually report many cases of BU and access to the centers could be a challenge especially during the rainy season due to the poor state of roads. FGDs revealed that since treatment centers are relatively far away from other health districts and considering the poor state of roads, patients preferably seek treatment elsewhere, which include herbalists/witchdoctors.

### Attitude towards BU patients

The level of acceptance of BU patients was high, since 70.3% of respondents regarded them as normal people. However, their attitude towards the patients was average because only 41.6% of them would allow their family members to interact with them and only 57.5% thought they should be allowed to go to school or public places while 57.8% attributed traditional beliefs to BU. This was confirmed by the FGDs and interview with BU patients. While some respondents demonstrated rejection and discriminatory attitudes towards BU victims, some showed positive attitudes. As shown by respondents and FGDs, positive attitudes were particularly demonstrated by those who were relatives of BU patients or those who understood the disease. The associated stigma even caused some patients to have marital problems. Reasons given for rejection of the disease victims were fear of communicability of the disease, disgusting nature of the wound and the repulsive smell, and fetish beliefs which attributed the disease to mystical causes.

Attitude and perception were influenced by socio-demographic factors of the respondents as seen from multiple logistic regression analysis without adjusting for other variables. Acceptance of patients by regarding them as normal people was significantly influenced by education, religion and occupation. Gender, education, and occupation significantly influenced the attitude of allowing one’s relatives to interact with BU patients. However, gender, occupation and time spent in the community were the important factors after unadjusted OR. There was no significant factor influencing the respondents’ attribution of BU to traditional beliefs. However unadjusted OR showed religion and education as important factors. Allowing BU patients to go to public places was influenced significantly by education, occupation and religion.

The importance of level of education in understanding a disease has been shown by Owusu-Sekyere *et al*. [32]. Education is a means of social change and helps in changing ones perception about the occurrence of a phenomenon. In this study, level of education played an important role in the acceptance of BU patients by community members. The majority of respondents had a level of education at the secondary and above (Table 3). The strong association between education and perception was demonstrated in the unadjusted OR at 95% CI (Table 4). Our findings corroborate the reports of Renzaho *et al*. [29] and Owusu-Sekyere *et al*. [32] which revealed education to be a determinant of perception towards BU patients. In a similar way, there was an association between occupation and attitude toward BU patients. Farmers were less likely to think that BU patients should be allowed to go to school and other public places, less likely allow their relations to interact with patients and less likely thought that patients should be regarded as normal people. Contrary to the report of Renzaho *et al*. [29] in which BU patients were not discriminated and stigmatized, our study shows rejection of these people by some members of their community.

## Conclusion

Our study clearly indicates that there is still a wide gap in the public awareness on BU disease in the study area. Although participants had a high level of awareness of the disease, our study revealed misconceptions about its etiology and transmission which greatly influences treatment seeking behavior and attitude of the community members towards BU patients, and could also expose them to infection. Proper community education is therefore urgently needed to correct the misconceptions about BU in study sites for any public health intervention targeting disease eradication to be successful.

## Supporting Information

S1 Table Biographic information of FGD participants (DOCX)

S1 Text Questionnaire Administered to Community Household Heads (PDF)
