# Supplementary material for: A Community Based Study on the Mode of Transmission, Prevention and Treatment of Buruli Ulcers in Southwest Cameroon: Knowledge, Attitude and Practices
Source: PLoS One. 2016 May 26;11(5):e0156463. doi: 10.1371/journal.pone.0156463 (PMC4881961; doi:10.1371/journal.pone.0156463)
Supplement: S1 Table — (DOCX) [file pone.0156463.s001.docx]

| **Participant code** | **Sex** | **Age** | **Health District** |
| --- | --- | --- | --- |
| 1E | M | 55 | Ekondo-titi |
| 2E | M | 46 | Ekondo-titi |
| 3E | F | 45 | Ekondo-titi |
| 4E | M | 50 | Ekondo-titi |
| 5E | M | 48 | Ekondo-titi |
| 6E | F | 37 | Ekondo-titi |
| 7E | F | 48 | Ekondo-titi |
| 8E | M | 44 | Ekondo-titi |
| 1Mb | F | 39 | Mbonge |
| 2Mb | F | 54 | Mbonge |
| 3Mb | M | 33 | Mbonge |
| 4Mb | F | 48 | Mbonge |
| 5Mb | M | 37 | Mbonge |
| 6Mb | M | 60 | Mbonge |
| 7Mb | M | 43 | Mbonge |
| 8Mb | F | 45 | Mbonge |
| 9Mb | M | 51 | Mbonge |
| 10Mb | M | 37 | Mbonge |
| 1M | F | 42 | Muyuka |
| 2M | M | 37 | Muyuka |
| 3M | M | 44 | Muyuka |
| 4M | M | 52 | Muyuka |
| 5M | M | 55 | Muyuka |
| 6M | F | 62 | Muyuka |
| 7M | M | 55 | Muyuka |
| 8M | M | 63 | Muyuka |
| 9M | F | 40 | Muyuka |

S1 Table: Biographic information of FGDs participants
