# Supplementary material for: A Community Based Study on the Mode of Transmission, Prevention and Treatment of Buruli Ulcers in Southwest Cameroon: Knowledge, Attitude and Practices
Source: PLoS One. 2016 May 26;11(5):e0156463. doi: 10.1371/journal.pone.0156463 (PMC4881961; doi:10.1371/journal.pone.0156463)
Supplement: S1 Text — (PDF) [file pone.0156463.s002.pdf]

Questionnaire for Community members

To obtain the required information, a questionnaire has been designed for you to complete. Your voluntary participation in the study is very vital as information provided will contribute to a better management of Buruli Ulcer cases. You are kindly requested to be honest in your responses and rest assured that information you will give is going to be treated with the strictest confidentiality. Thank you very much for your kind response.

Personal Information

Study ID : ____

Today's date: _______________

Village of Residence :____

Tel : ___________________________

AGE : _______

Sex : (a) Male (b) Female

Instructions: please tick (√) the letter that corresponds to the appropriate response

Background

1. What is your highest level of education?

   (a) Primary (b) Secondary and High School (c) University (d) No education

2. What is your marital status?

   (a) Married (b) Single (c) Divorced (d) Widowed

3. How long have you lived in the above address?

   (a) Less than one year (b) Between 1 and 5 years (c) More than 5 years

4. What is your occupation?

   (a) Farming (b) Trading (c) Professional/admin (d) Other

5. What is your religion?

   (a) Christian (b) Muslim (c) Traditional (d) Other

Knowledge about Buruli Ulcer (BU)

6. Do you know what BU is?

(a) Yes (b) No

7. Do you have any local name you call it?

(a) Yes (b) No

8. If yes, state the name, and why do you call it that way?

____

____

____

9. What causes BU?

____

____

10. Do you think BU is a health Problem?

(a) Yes (b) No

11. Indicate a means by which this disease can be transmitted or how one can get infected (*tick*)

☐Worms in ponds ☐Poor hygiene ☐Witchcraft ☐Bite by mut mut fly ☐Bite by an insect

☐ Snake bite ☐ Bacterial infection ☐ Don't know

12. Do you believe BU can be transmitted from one person to another?

(a) Yes (b) No

13. Do you think BU can be treated?

(a) Yes (b) No

14. If yes, which in your opinion is the best method that can be used to treat BU?

(a) Hospital (b) Herbalist/witch Doctor (c) Prayer (d) other (*please specify below*)

____

____

15. Can getting infected with this disease be prevented?

(a) Yes (b) No

16. If yes tick the best way by which it can be prevented

☐ Wearing protective clothes and shoes ☐ avoid dirty water and swamps

☐ Proper hygiene and sanitation ☐ Don't know

Attitude and Practices

17. Have you ever seen anyone with BU?

(a) Yes (b) No

18. If yes, how did/do you interact with him/her?

(a) Freely (b) with restrictions (c) No interaction

19. Give reasons

____

____

20. What is your relationship with the patient?

(a) Family member (b) Friend (c) Not related

21. Do you think a person with BU can be regarded as a normal person in the society?

(a) Yes (b) No

22. Will you allow your child or family member to interact freely with a BU patient?

(a) Yes (b) No

23. Are there any traditional beliefs attributed to his illness in your community?

(a) Yes (b) No

24. If yes what kind of beliefs?

(a) Curse (b) Possessed (c) Other (*please specify below*)

____

____

25. Should BU patients be allowed to go to school or to public places where they will meet with uninfected people?

(a) Yes (b) No

END
